# Prevalence and associated factors of zinc deficiency among pregnant women and children in Ethiopia: a systematic review and meta-analysis

**DOI:** 10.1186/s12889-019-7979-3

**Published:** 2019-12-11

**Authors:** Kidanemaryam Berhe, Freweini Gebrearegay, Hadush Gebremariam

**Affiliations:** Department of Nutrition and Dietetics, School of Public Health, College of Health Sciences, Mekelle University, Mekelle, Tigray Ethiopia

**Keywords:** Zinc deficiency, Children, Pregnant women, Ethiopia

## Abstract

**Background:**

Pregnant women and children are the most vulnerable groups to zinc deficiency. Despite the presence of few primary studies, studies that could provide strong evidence that would help policymakers to develop appropriate interventional strategies in addressing zinc deficiency among pregnant women and children are limited in Ethiopia. Therefore, the aim of this systematic review and meta-analysis was to estimate the national pooled prevalence and associated factors of zinc deficiency among pregnant women and children.

**Methods:**

We searched Pub Med, Scopus, CINAHL, Google Scholar, and Google for studies reported on zinc deficiency and associated factors among pregnant women and children. Search terms were ‘zinc deficiency’, ‘zinc status’, ‘associated factors’, ‘children’, ‘pregnant women’, and ‘Ethiopia’ using the boolean operators ‘OR’ or ‘AND’. Searches were using English language. A preferred reporting item for systematic reviews and meta-analysis (PRISMA) checklist was used. Two authors independently reviewed the studies. The effect sizes of the meta-analysis were the prevalence of zinc deficiency and adjusted odds ratio (AOR) of the associated factors. Finally, the Comprehensive Meta-Analysis (CMA) version 3.3.07 was used for statistical analysis by applying the random-effects model and publication bias was assessed using funnel plots and Egger’s test.

**Results:**

Thirteen studies (7 among pregnant women having total participants of 2371 pregnant women and 6 among children with total participants of 5154 children) were included in this systematic review and meta-analysis. Using the random-effects model, the pooled prevalence of zinc deficiency was 59.9% (95%CI: 51.9, 67.7%) and 38.4% (95%CI: 28.6, 49.4) among pregnant women and children, respectively. The associated factors for zinc deficiency among pregnant women were coffee intake (adjusted odds ratio (AOR) =1.76), low intake of animal source foods (AOR = 2.57), and inadequate diet diversity (AOR = 2.12).

**Conclusion:**

Overall, zinc deficiency among pregnant women and children is a significant public health concern in Ethiopia. Promoting dietary modification to enhance the bioavailability of zinc, improving diet diversity, and consuming animal source foods would help in alleviating and/or minimizing the problem among the target groups. Zinc supplementation could also be considered for pregnant women and children.

## Plain English summary

Zinc deficiency is a worldwide problem which affects around 17% of the world’s population but mainly affects sub-Saharan Africa and South Asia. Children and pregnant women are the most risk groups for zinc deficiency. To take action on zinc deficiency it is important to have evidence on the national prevalence and associated factors of zinc deficiency among the high-risk groups (children and pregnant women). Checklist was used during the systematic review and meta-analysis and comprehensive search of studies up to May 30, 2019, was made from different search engines. The primary outcome of this systematic review and meta-analysis was the prevalence of zinc deficiency among pregnant women and children. A total of 13 studies (7 for pregnant women and 6 for children) were included in this systematic review and meta-analysis. A total of 2371 pregnant women and 5154 children were included in this systematic review and meta-analysis. The prevalence of zinc deficiency was 59.9%& and 38.4% among pregnant women and children, respectively. The associated factors for zinc deficiency among pregnant women were coffee intake, low intake of animal source foods and inadequate diet diversity. No associated factor was identified for zinc deficiency among children. From this, it is possible to conclude that zinc deficiency among children and pregnant women is a public health concern that needs attention and action. To solve zinc deficiency, it is important to consider a different approaches like diet modification, promoting consuming animal source foods, stoping or reducing coffe intake, and zinc supplementation.

## Background

Worldwide, zinc deficiency is a significant micronutrient deficiency like iron, iodine, and vitamin A deficiency states [1–3]. Its effects on the child’s health, growth, and development as well as on mother’s health were not fully recognized by the United Nations until 1997. After then, it was included as one of the priority micronutrient deficiencies in the third report of the World Nutrition Situation (WNS) [4]. Adequate serum zinc in pregnant women helps to have optimal health and wellbeing for both the woman and her growing fetus as it is very crucial for protein synthesis, cellular growth, cell differentiation immunity function, and growth and development especially for the fetus [5–7].

At least 17% of the world’s population is at risk of inadequate zinc intake with the highest risk occurring in sub-Saharan Africa and South Asia [5]. In 2011, zinc deficiency was accounted for about 116,000 child deaths and 20% perinatal mortality in the world (6). In Africa, zinc deficiency accounted for 14.4% of diarrheal deaths, 10.4% of malaria deaths and 6.7% of pneumonia deaths among children between 6 months and 5 years of age [7]. Zinc deficiency ranged from 9.5% in developed countries to 33.5% in developing countries [8].

Many observational studies have also shown that zinc deficiency is associated with congenital anomalies, fetal growth retardation, fetal death, impaired immune function, and impairment of learning and memory function. Furthermore, the linkage of zinc deficiency to a wide range of pregnancy-related complications such as prolonged labor, premature rupture of membrane, placental abruption, and infections have been reported in many studies [6, 7, 9, 10]. However, the level of evidence is not strong enough for policymakers to design appropriate interventional strategies and these consequences of zinc deficiency have not been supported by a meta-analysis of randomized control trials (RCTs). Zinc deficiency could be resulted due to many reasons including poor dietary zinc intake, increased loss, food insecurity, poor household food distribution, presence of fiber and/or phytate in diets, inappropriate food preparation and storage and infection [10]. Evidence showed that people who live in low and middle-income countries consume mainly plant-based diets which contain a high amount of phytate (a substance that inhibits the absorption of zinc), and diets based on starchy roots and/or tubers which are known to have low zinc content that can eventually result in zinc deficiency [5, 11, 12].

Micronutrient deficiencies are among the significant public health concerns in Ethiopia [11]. To address these problems, the government of Ethiopia has been taking different initiations including developing and implementing nutritional programs and establishing nutrition and food policy, which was developed recently [12, 13]. Despite the development of policy and program to tackle nutrition-related problems in the nation, evidences on the prevalence and associated factors of zinc deficiency among pregnant women and children are not strong enough to the level policymakers could develop evidence-based intervention strategies in the country except zinc supplementation for children who visit health facilities with the complain of diarrhea. There are no meta-analysis studies addressing zinc deficiency among children and pregnant women in Ethiopia that could give high-level evidence to policymakers. Therefore, this systematic review and meta-analysis was designed to address the gap and estimate the pooled prevalence and associated factors of zinc deficiency among the vulnerable groups (children and pregnant women) in Ethiopia.

## Methods

### Eligibility criteria and information sources

This systematic review and meta-analysis included studies conducted in Ethiopia with the objective of assessing prevalence of zinc deficiency and associated factors among pregnant women and/or children. Before including the studies in the final review, studies were assessed for inclusion criteria using the title, abstract and a full review of the studies. Preferred reporting items for systematic reviews and meta-analysis (PRISMA) checklist was used during the review [14]. Published articles, survey reports, and grey literature (one paper) [15] which were published or reported in English were considered in this meta-analysis. Due to the limited number of studies conducted in Ethiopia, and to increase the comprehensiveness of the meta-analysis, all eligible studies published or reported till May 30, 2019, were included in this systematic review and meta-analysis. Articles retrieved from the databases were exported to Endnote version X6 to facilitate the article selection process and manage citation.

### Search and study selection

Studies were identified by searching electronic databases, and scanning reference lists of articles. PubMed, Scopus, CINAHL, Google Scholar, and Google were searched to find out articles for this systematic review and meta-analysis. Though the PubMed database is one of the most comprehensive sources of health studies in the world, its coverage is not complete; that is why we considered additional search databases to make our searching comprehensive and complete. Two authors performed the search activities independently using the following search terms to find all relevant studies in the search databases: ‘zinc deficiency’, ‘zinc status’, ‘micronutrient deficiencies’, ‘micronutrient status’, ‘micronutrient deficiency’, ‘prevalence’ or ‘magnitude of zinc deficiency’, ‘associated factors’, ‘risk factors’, ‘determinants’, ‘children’, ‘pregnant women’ and ‘Ethiopia’ separately and/or in combination using the Boolean operator like ‘OR’ or ‘AND’.

### Data collection process and data items

A predefined data extraction format was used to collect information. Name of the author(s), publication year, region, study design, study population, sample size, and response rate were some of the information extracted for both the prevalence and associated factors. Prevalence of zinc, time point measured and reported, cut-offs of serum zinc, subgroup analysis if any, and appropriateness of methods were collected for zinc prevalence and similarly, name of associated factor, time points measured and reported, adjusted odds ratio, appropriateness of methods, and controlling of confounding factors were collected for the associated factors [Additional file 1].

### Assessment of quality of the studies

The studies were assessed using the criteria proposed in the protocol called Modified Newcastle-Ottawa quality assessment scale for non-randomized studies [16]. Two authors independently assessed the quality of the studies and in the case where the two authors didn’t agree, it was resolved by discussion and involvement of the third author. Parameters such as sampling strategy, inclusion/exclusion criteria, sample size, cut-offs for zinc status, and statistical models were assessed to identify the associated factors. A total score of 9 stars was considered as maximum and zero as a minimum [Additional file 2]. A study was considered high quality if it scored 7 and above. All the studies included in this systematic review and meta-analysis have scored 7 and above.

### Summary measures

The primary outcome of this systematic review and meta-analysis was the prevalence of zinc deficiency among pregnant women and children. Guidelines on the assessment of population zinc status were published by the World Health Organization (WHO), the United Nations Children’s Fund (UNICEF), the International Atomic Energy Agency, and the International Zinc Nutrition Consultative Group (IZiNCG) in 2007(10). Recommended indicators included plasma or serum zinc concentration and dietary zinc intake through the use of 24-h recalls or weighed food records. The stunting prevalence among children less than 5 years of age was suggested as a proxy of the likely risk of zinc deficiency in a population but serum/plasma zinc level is the best available biomarker of the risk of zinc deficiency [10]. In this systematic review and meta-analysis, the risk of zinc deficiency which was considered as high and public health concern if the prevalence of low serum zinc concentration is > 20% or the prevalence of inadequate zinc intake is > 25%. Stunting prevalence 20% or above among preschool children reflects zinc deficiency [17, 18]. The second outcome of this systematic review and meta-analysis was the associated factors of zinc deficiency. The effect sizes of this systematic review and meta-analysis were the prevalence of zinc deficiency and the adjusted odds ratios (AORs) of the associated factors. Factors that were significant in at least two studies were included in this meta-analysis.

### Statistical methods and analysis

A software called Comprehensive Meta-Analysis (CMA) version 3.3.07 (November 20, 2014) was used for statistical analysis. Due to the heterogeneity nature of the studies, the random effect model was used as a method of analysis. Subgroup analysis by age was performed for children but no subgroup analysis for pregnant women due to a limited number of studies. The factors associated with zinc deficiency were identified by looking at the adjusted ORs and 95% CIs reported in each study. The adjusted odds ratios of the associated factors were analyzed using separate categories of meta-analysis. The effect sizes were reported with their 95% confidence intervals (CIs). The findings of the systematic review and meta-analysis were presented using tables, graphs, and texts.

### Publication bias and heterogeneity

Publication bias was assessed using funnel plots and Egger’s test [19]. In the funnel plot, the symmetry was assessed and Egger’s test a *p*-value< 0.05 was used to declare the statistical significance of publication bias. I^2^ test statistics were used to check the heterogeneity of studies. I^2^ statistics described the total variation across studies. I^2^ test statistics of < 50, 50–75% and > 75% was declared as low, moderate and high heterogeneity respectively [20].

## Results

### Study selection

**Fig. 1:**
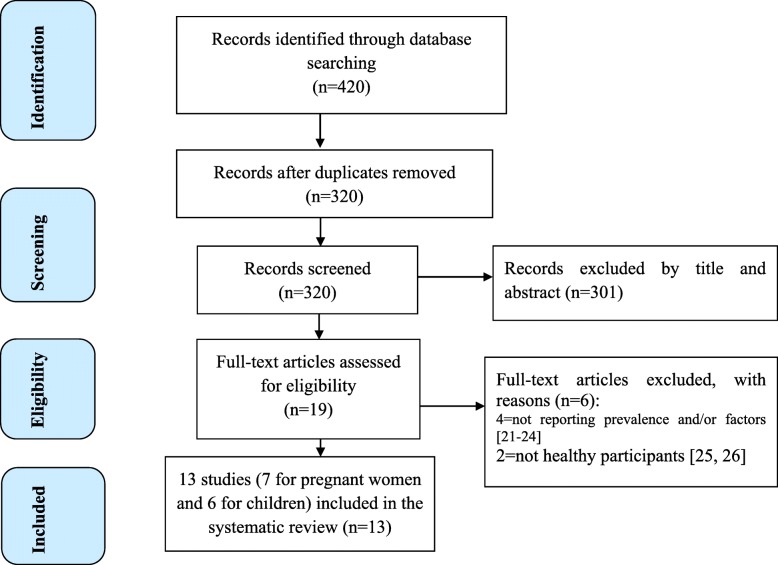
Flow diagram of the studies included in the systematic review and meta-analysis of zinc deficiency among pregnant women and children in Ethiopia, 2019

In the initial search, we found a total of 420 records from different electronic search databases. After screening the title and abstract of the studies, 301 records were excluded from this analysis. Again, we excluded 100 duplicate records and finally we left with 19 records. After assessing the full texts of the 19 records for their eligibility, 6 records [21–26] were further excluded by the exclusion criteria. Finally, 13 studies, 7 for pregnant women [15, 27–32] and 6 for children [11, 33–37], were found to be eligible for this systematic review and meta-analysis (Fig. 1). Data from the study conducted by Ethiopia public health institute (EPHI); among the studies included for children, the prevalence of zinc deficiency for under 5 children and for above 5 children were extracted separately because the prevalence of zinc deficiency for these two groups was separately estimated in the paper.

### Characteristics of the studies

All the studies included in this systematic review and meta-analysis were cross-sectional studies. A total of 2371 pregnant women and 5154 children were included in this systematic review and meta-analysis. The sample size among pregnant women ranged from 99(26,28) to 750(35) and among children ranged from 100 [37] to 1569 [11]. From the studies conducted among pregnant women, 3 studies were conducted in the south nation, nationalities, and peoples (SNNP) of Ethiopia, 2 studies were conducted in Amhara regional state), 1 in Gambela regional state and 1 in Addis Ababa (the capital city of Ethiopia). Studies included in this systematic review showed that the prevalence of zinc deficiency among pregnant women in Ethiopia ranged from 39.5% [30] to 76% [23] (Table 1). In five studies, zinc deficiency was defined as per the recommendation of International Zinc Nutrition Consultative Group (IZiNCG) i.e. serum zinc < 56 μg/dl during the first trimester, or < 50 μg/dl during the second or third trimester [38] but zinc deficiency for two studies was defined at < 75 μg/dl [30, 35]. The laboratory analysis approaches used to determine participants’ serum level of zinc by each study included in this systematic review and meta-analysis are described in an additional file [Additional file 3].

**Table 1:** Prevalence of zinc deficiency among pregnant women, from individual studies, Ethiopia, 2019

Name of author	Region	Study design	Sample size	Response rate	Cutoffs used (μg/dl)	Prevalence	Quality score
Gebremedhin S et al 2011	SNNP	Cross-sectional	750	93.3%	Serum zinc < 56 in the 1^st^ TM and < 50 during the 2^nd^ or 3^rd^ TM	53%	9
Stoecke BJ et al 2009	SNNP	Cross-sectional	99	100%	Plasma Zinc< 50	76%	7
Afework K et al 2008	Amhara	Cross-sectional	375	100%	Serum zinc level <75	66.7%	7
Regassa K 2017	Addis Ababa	Cross-sectional	403	100	Serum zinc level <75	39.5%	7
Kumera G K et al 2015	Amhara	Cross-sectional	377	96.5 %	Serum zinc level < 56 in the 1^st^ TM and <50 during 2^nd^ and 3^rd^ TM	57.4 %	8
Rosalind S et al 2008	SNNP	Cross-sectional	99	100%	Plasma zinc <50	74%	7
Mekonen A 2016	Gambela	Cross-sectional	268	100%	Serum zinc < 56 during the 1^st^ TM and < 50 during the 2^nd^ or 3^rd^ TM	55.3%	8

*TM* trimester, *SNNP* south nation, nationalities, and peoples

Out of the seven studies included in this systematic review and meta-analysis, four studies identified associated factors for zinc deficiency among pregnant women (Table 2) but only one study conducted among children reported associated factors (consumption of legumes and nuts with adjusted odds ratio (AOR) =0.37, consuming meat and fish with AOR = 0.22 and low diet diversity score with AOR = 1.42) for zinc deficiency.

**Table 2:** Associated factors of zinc deficiency among pregnant women, from individual studies, Ethiopia, 2019

Author (s)	Associated factors	AOR(95%CI)
Gebremedhin S et al. 2011	No intake of animal source foods	2.51 (1.7,3.72)
	Age (≥35 years)	2.18 (1.25,3.63)
	Illiterate mothers	1.7 (1.09,2.6)
	Inadequate DDS	2.57 (1.57,4.18)
	Food insecurity	5.07 (3.67,6.99)
	Coffee intake	1.41 (1.06,1.84)
Regassa K 2017	No intake of animal source foods	2.11 (1.3,3.42)
	Drinking coffee	2.12 (1.39,3.42)
	Elevated C-reactive protein	2.48 (1.45,4.24)
Kumera G et al. 2015	Living in a rural area	1.92 (1.04,3.56)
	Too close birth	3.97 (1.3,12.13)
	No intakes of animal origin foods	2.29 (.35,3.89)
	Inadequate DDS	2.09 (1.24,3.51)
	No nutrition education	1.78 (1.1,2.86)
	Low serum albumin	2.55 (1.4,4.63)
	Presence of intestinal parasitic infection	2.6 (1.49,4.54)
Mekonen A 2016	No nutrition education	2.4 (1.01,5.74)
	3rd trimester	3.76 (1.49,9.49)
	No intake of animal-sourced foods	3.05 (1.3,7.07)
	Inadequate DDS	3.59 (1.45,8.96)

*DDS* diet diversity score, *AOR* adjusted odds ratio, *CI* confidence interval

From the studies conducted among children, 3 studies were conducted in Amhara regional state, 2 studies were conducted at the national level and 1 study was conducted at two agro-ecological zones of rural Ethiopia (Babile (Oromia), Enderta and Hintalo wajirat (Tigray)). The studies included in this systematic review and meta-analysis for children showed that the prevalence of zinc deficiency among children ranged from 35% [11] to 67.3% [34]. Only in one study, zinc deficiency among children was low (12.5%) [35]. In these studies, the cut-offs of serum zinc levels were different from the study to study. Serum zinc level < 65 μg/dl was used for less than 10 years old children, and < 70 μg/dl for 10 years and above children. This cutoffs of serum zinc levels are similar to the recommendation of International Zinc Nutrition Consultative Group (IZiNCG) [38], but only one study used serum zinc level of < 70 μg/dl for all children with the age ranged from 6 months to 14 years (Table 3).

**Table 3:** Prevalence of zinc deficiency among children, from individual studies, Ethiopia, 2019

Name of author	Region	Study design	Age of children	Sample size	Response rate	Cutoffs used (μg/dl)	Prevalence	Quality score
Zaida H et al. 2014	Amhara	Cross-sectional	4–15 years	764	80.9%	SerumZinc< 65 for children< 10 years and < 70 for children> 10 years	12.5%	8
Bemnet A et al. 2012	Amhara	Cross-sectional	10–14 years	100	100%	Serum zinc < 75	47%	7
Roba K et al. 2018	Babile (Oromia), Enderta and Hintalowajirat (Tigray)	Cross-sectional	6–23 months	162	100%	Low serum zinc< 65	67.3%	7
Masresha T et al. 2019	National level	Cross-sectional	6–59 months	1176	100%	Serum zinc< 65	28%	8
Adamu B et al. 2015	Amhara	Cross sectional	6–60 months	240	100%	Serum zinc level < 65	57.1%	8
EPHI 2016	National level	Cross sectional	6–59 months	1143	100%	Serum zinc level < 70	35%	8
			5–14 years	1569	100%	Serum zinc level < 70	36%	8

*EPHI* Ethiopia Public Health Institute

### The pooled prevalence of zinc deficiency among the pregnant women and children

**Fig. 2:**
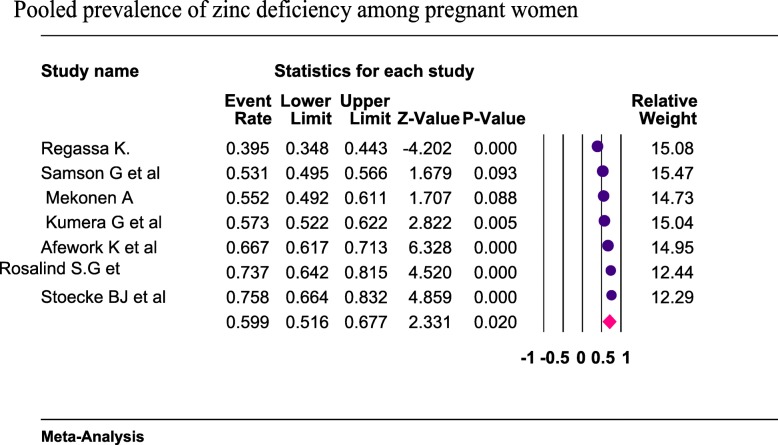
Forest plot for the pooled prevalence of zinc deficiency among pregnant women in Ethiopia, 2019

Seven studies were included in the analysis to estimate the pooled prevalence of zinc deficiency among pregnant women. Using the random-effects model, the pooled prevalence of zinc deficiency among pregnant women was 59.9% (95%CI: 51.9, 67.7%) (Fig. 2). The heterogeneity among the seven studies used to estimate the pooled prevalence of zinc deficiency among pregnant women was very high (I^2^ = 93.3%; *p* < 0.001).

**Fig. 3:**
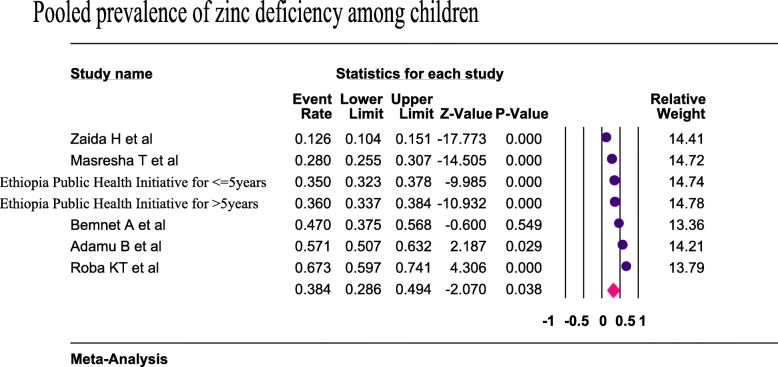
Forest plot for the pooled prevalence of zinc deficiency among children in Ethiopia, 2019

**Fig. 4:**
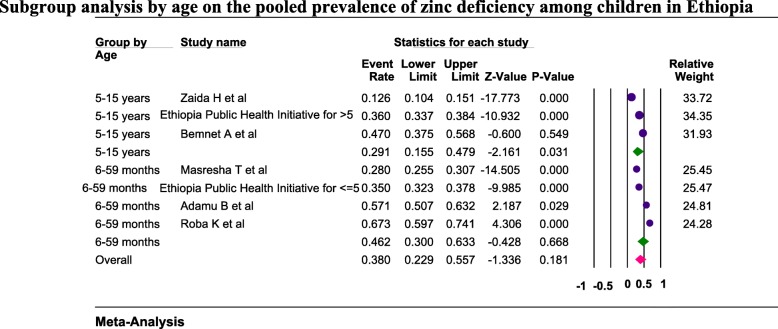
Subgroup analysis by age on the pooled prevalence of zinc deficiency among children in Ethiopia, 2019

Six studies were included to estimate the pooled prevalence of zinc deficiency among children. Using the random-effects model, the pooled prevalence of zinc deficiency among children was 38.4% (95%CI: 28.6, 49.4) (Fig. 3). Heterogeneity among the studies used to estimate the pooled prevalence of zinc deficiency among the children was very high (I^2^ = 97.9%; p < 0.001). The subgroup analysis by age was computed for children and the pooled prevalence was higher (46.2%) among 6–59 months old children (Fig. 4).

### The associated factor for zinc deficiency among pregnant women

Four studies were included in the analysis of associated factors for zinc deficiency among pregnant women [15, 29, 30, 32]. For a factor to be included in the meta-analysis, it has to be significant in at least two studies. Three associated factors were included in the meta-analysis: coffee intake [Additional file 4], low intake of animal source foods [Additional file 5] and inadequate diet diversity [Additional file 6]. The pooled adjusted odds ratios ranged from 1.76 to 2.57. Heterogeneity was observed among all studies included in the analysis of these associated factors (Table 4). For the children, no associated factor was mentioned in at least two studies thus no meta-analysis for associated factor among the studies for children was done.

**Table 4:** Summary of meta-analysis for the associated factors of zinc deficiency among pregnant women in Ethiopia, 2019

S.no	Associated factors	Number of studies	AOR (95%CI)	*P*-value	Heterogeneity			Egger’s test (*p*-value)
					Q-value	*P*-value	I^2^	
1	Coffee intake	2	1.76(1.05,2.92)	0.026	3.99	0.046	74.9	0.98
2	Low intake of animal source foods	4	2.57(1.8,3.66)	<0.001	8.03	0.045	62.7	0.96
3	Inadequate diet diversity	3	2.12 (1.28,3.53)	0.003	8.71	0.013	77	0.71

*AOR* adjusted odds ratio, *CI* confidence interval

### Publication bias

**Fig. 5:**
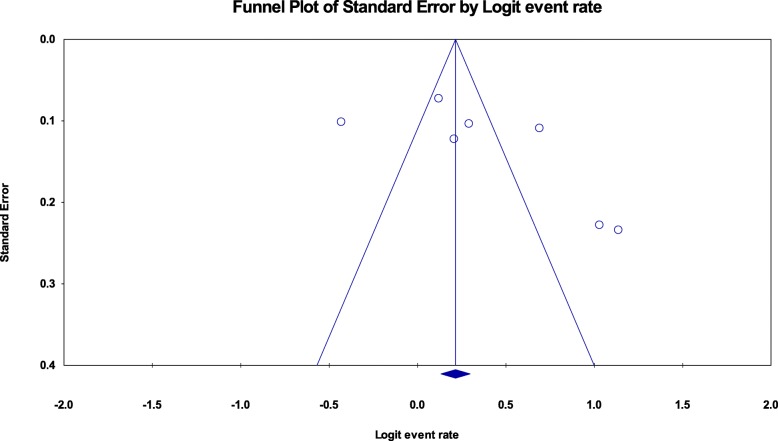
Funnel plot for assessing publication bias among studies included to estimate the pooled prevalence of zinc deficiency among pregnant women in Ethiopia, 2019

**Fig. 6:**
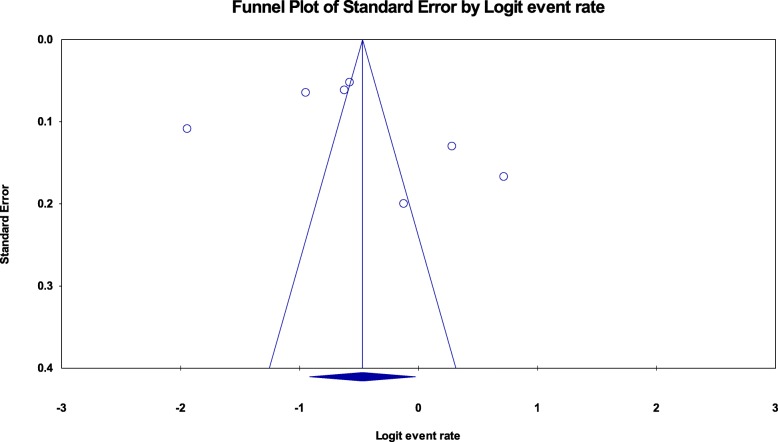
Funnel plot for assessing publication bias among studies included to estimate the pooled prevalence of zinc deficiency among children in Ethiopia, 2019

Publication bias was assessed using the funnel plot for asymmetry and Egger’s test. For the studies included to estimate the pooled prevalence of zinc deficiency among pregnant women and children, the funnel plots were a little bit asymmetry (Fig. 5 seems a little bit tilted to the right and Fig. 6 seems tilted to the left, respectively) but Egger’s test was not significant (*p*-value = 0.164, 0.53 respectively) (Table 3, above).

## Discussion

Generally, there are limited studies available in Ethiopia on the prevalence and associated factors of zinc deficiency among pregnant women and/or children. The assessment of zinc status may be hindered by a lack of standard biomarker, accepted guidelines on which indicators to use, how to carry out the assessment, and how to interpret the results. Similarly, the lack of good biomarkers can affect identifying the risk factors for zinc deficiency [10]. The random-effects model was used for meta-analysis due to the likelihood of significant heterogeneity among the studies. The estimated pooled prevalence of zinc deficiency among pregnant women was 59.9% (95CI%: 51.6, 67.7%). The prevalence in this systematic review and meta-analysis was lower as compared to the prevalence reported from Cameron (82%) [39] but higher when compared with the prevalence reported from Nigeria (28.1%) [40]. The difference could be due to socio-economic and methods differences. The finding of this systematic review and meta-analysis implied that zinc deficiency among pregnant women is a public health concern in Ethiopia. Therefore, zinc intervention strategies should be considered to address zinc deficiency. Zinc supplementation could be helpful because evidence showed that zinc supplementation for pregnant women can reduce adverse birth outcomes in low-income settings [6].

In this systematic review and meta-analysis, the associated factors for zinc deficiency among pregnant women were coffee intake, low intake of animal source foods and inadequate dietary diversity. Populations in the developing countries including Ethiopian consume limited animal products (excellent source of zinc) and they usually consume plants or cereal meals which contains non-digestible constituents such as phytates, dietary fibers, and lignin that can bind zinc in ways that inhibit its absorption thereby increased risk of zinc deficiency [9, 41]. About 100% of women in developing countries have usual intakes of zinc considered inadequate to meet the normative needs of pregnancy [42]. Similarly, diet diversity score among pregnant women in Ethiopia is low but the drinking of coffee, which contains caffeine that inhibits the absorption of zinc, is common [43]. These factors were identified based on a few studies, thus it needs caution in interpretation and generalizing of the findings.

Six studies assessed the prevalence of zinc deficiency among children in Ethiopia and the estimated pooled prevalence was 38% (95%CI: 22.9, 55.7%). The subgroup analysis by age revealed that zinc deficiency was high (48.2%) among the under 5 years old children as compared to 5–15 years old children (29.1%). In this systematic review and meta-analysis, zinc deficiency among children was a public health concern. This prevalence (38%) is higher as compared to zinc deficiency prevalence among children in Nepal [44], Srilanka [45], Nigeria [40], and Afghanistan [46] which was 21, 5.1, 20, and 15.1% respectively but it is lower as compared to the finding from Cameron (83%) [39]. This difference could be because of the difference in child nutritional practices, socio-economic, and methods in conducting the studies. The studies included in this systematic review and meta-analysis didn’t represent all regions (nine) and city administrations (two) of Ethiopia but stunting prevalence was estimated in all regions and city administrations during the Ethiopia Demographic and Health Survey (EDHS) which is conducted every five years. In the recent survey report (EDHS 2016) except in Addis Ababa, stunting prevalence ranged from 23.5% (Gambela region) to 46.3% (Amhara region). At the national level stunting prevalence was 38% [47] which is inline with the pooled prevalence of zinc deficiency among children. This shows that zinc deficiency is a public health concern of all regions and city administrations in Ethiopia which needs attention and action. The role of zinc in the linear growth of children is due to the contribution of zinc in more than 300 enzyme actions, protein synthesis, gene expression, and bone growth [48, 49].

Diarrheal diseases can exacerbate zinc deficiency due to intestinal losses [6]. In 2016, about 12% of under 5 years old children had diarrheal diseases in Ethiopian. The low coverage of safe water supply (57%) and low proper hand-washing practices (7%) in the communities may contribute to the high burden of diarrheal diseases in Ethiopia [9]. Evidence showed that zinc supplementation for children decreases morbidity and mortality rates of diseases (e.g. diarrhea, pneumonia, and malaria) [41]. Similarly, zinc supplementation resulted in a 9% reduction of all-cause child mortality and increases linear growth in under five years old children [6].

As a strength, this systematic review and meta-analysis used a comprehensive search strategy and more than two reviewers were involved in each step of the review process. Moreover, the PRISMA guideline was strictly followed during the review process. But, this systematic review and meta-analysis has limitations as well; the number of studies included was small, all the regions and city administrations in Ethiopia were not represented in the studies included in this systematic review and meta-analysis thus caution is required in interpretation and generalizing of the findings. All the studies were cross-sectional which could affect the temporal relationship and associated factors for zinc deficiency among children were not found. In addition, many studies did not report how the specimens were processed or whether trace element free laboratory supplies were used to reduce the risk of contamination. Contamination from exogenous sources of zinc can change the results [17, 41, 48, 50–52]. Sub-group analysis using different variables (e.g. region) was not possible due to a limited number of studies. Due to the lack of established and sensitive biomarkers, measuring prevalence based on serum zinc could underestimate the extent of zinc deficiency. Lastly but not least two studies from the studies among the pregnant women and one study from the children didn’t apply the recommendations of International Zinc Nutrition Consultative Group (IZiNCG) for the cut-off serum zinc level which can underestimate zinc deficiency.

## Conclusion

Overall, zinc deficiency among pregnant women and children is a significant public health concern in Ethiopia. Promoting dietary modification to enhance the bioavailability of zinc, improving diet diversity, and consuming animal source foods would help in alleviating and/or minimizing the problem among the target groups. Zinc supplementation could also be considered for pregnant women and children.

## Supplementary information

**Additional file 1.** Format for extraction of data for systematic review and meta-analysis of prevalence and associated factors of zinc deficiency among pregnant women and children in Ethiopia, 2019.

**Additional file 2.** Filled checklist for quality assessment of the included studies in the systematic review and meta-analysis of prevalence and associated factors of zinc deficiency among pregnant women and children in Ethiopia, 2019.

**Additional file 3.** Table that shows the laboratory analysis approaches used to determine serum zinc level of the study participants of the studies included in the systematic review and meta-analysis of prevalence and associated factors of zinc deficiency among pregnant women and children in Ethiopia, 2019.

**Additional file 4.** Forest plot for coffee intake and zinc deficiency among pregnant women in Ethiopia, 2019.

**Additional file 5.** Forest plot for low intake of animal source foods and zinc deficiency among pregnant women in Ethiopia, 2019.

**Additional file 6.** Forest plot for inadequate diet diversity and zinc deficiency among pregnant women in Ethiopia, 2019.

## Data Availability

All data regarding this systematic review and meta-analysis are contained and presented in this systematic review and meta-analysis document.

## Abbreviations

AOR: Adjusted Odds Ratio
CI: Confidence Interval
CINAHL: Cumulative Index to Nursing and Allied Health Literature
DDS: Diet Diversity Score
EDHS: Ethiopian Demographic and Health Survey
IZiNCG: International Zinc Nutrition Consultative Group
PRISMA: Preferred Reporting Items for Systematic Review and Meta-Analysis
SNNP: South Nation, Nationalities and Peoples of Ethiopia
UNICEF: United Nation Children’s Fund
WHO: World Health Organization
