# Supplementary material for: Prevalence and associated factors of zinc deficiency among pregnant women and children in Ethiopia: a systematic review and meta-analysis
Source: BMC Public Health. 2019 Dec 11;19:1663. doi: 10.1186/s12889-019-7979-3 (PMC6907210; doi:10.1186/s12889-019-7979-3)
Supplement: Supplementary file 1 — Additional file 1. Format for extraction of data for systematic review and meta-analysis of prevalence and associated factors of zinc deficiency among pregnant women and children in Ethiopia, 2019. [file 12889_2019_7979_MOESM1_ESM.docx]

**Data extraction formats**

Title of the systematic review and meta-analysis: prevalence and associated factors of zinc deficiency among pregnant women and children in Ethiopia, 2019.

1. **General information**

| Date form completed |  |
| --- | --- |
| Data extractor |  |
| Title of the paper that data are extracted from |  |
| Name of author/s |  |
| Year of study |  |
| Study area/Region |  |
| Any important information |  |

1. **Study eligibility**

| Type of study |  |
| --- | --- |
| Population description |  |
| Types of outcome measures (zinc deficiency, associated factors) |  |
| Decision with reasons for either inclusion or exclusion |  |
| Any important information |  |

No need to proceed if the study is excluded from the review

1. **Methods**

| Aim of study |  |
| --- | --- |
| Study design |  |
| Sampling techniques |  |
| Study start date |  |
| Study end date |  |
| Any important information |  |

1. **Participants**

| Sample size |  |
| --- | --- |
| Study population |  |
| Any important information |  |

1. **Outcomes**

| - 1. Prevalence |  |
| --- | --- |
| Prevalence of zinc deficiency |  |
| Time points measured |  |
| Time points reported |  |
| Outcome definition (cut offs for serum zinc level) |  |
| Is tool validated(Yes/No/Unclear/Not mentioned) |  |
| - 1. Associated factor |  |
| Name of associated factor |  |
| Time points measured |  |
| Time points reported |  |
| Adjusted odds ratio and its 95% confidence interval |  |
| Any important information |  |

1. **Results**

| - 1. Prevalence |  |
| --- | --- |
| Subgroup prevalence if any e.g. by age |  |
| Results |  |
| Response rate |  |
| Any other results reported |  |
| Statistical methods used and appropriateness of these methods |  |
| Any additional information |  |
| - 1. Associated factors |  |
| Name of associated factors |  |
| Results |  |
| Response rate |  |
| Any other results reported |  |
| Statistical methods used and appropriateness of these methods |  |
| All systematic and random error adjusted (e.g. confounding factor) |  |

1. **Strength and limitation**

| Strength |  |
| --- | --- |
| Limitation |  |
| Any important information |  |

1. **Conclusion and other information**

| Key conclusions of study authors |  |
| --- | --- |
| Any important information |  |
