# Supplementary material for: Prevalence and associated factors of zinc deficiency among pregnant women and children in Ethiopia: a systematic review and meta-analysis
Source: BMC Public Health. 2019 Dec 11;19:1663. doi: 10.1186/s12889-019-7979-3 (PMC6907210; doi:10.1186/s12889-019-7979-3)
Supplement: Supplementary file 2 — Additional file 2. Filled checklist for quality assessment of the included studies in the systematic review and meta-analysis of prevalence and associated factors of zinc deficiency among pregnant women and children in Ethiopia, 2019. [file 12889_2019_7979_MOESM2_ESM.docx]

Quality assessment of the study included in the meta-analysis of prevalence and associated factors of zinc deficiency among pregnant women and children in Ethiopia

| **Scales** | | **Studies** | | | | | | | | | | | | |
| --- | --- | --- | --- | --- | --- | --- | --- | --- | --- | --- | --- | --- | --- | --- |
|  |  | Gebremedhin S et al 2011 | Stoecke BJ et al 2009 | Afework K et al 2008 | Regassa K 2017 | Gemechu K et al 2015 | Rosalind S et al 2008 | Ataguadil M 2016 | Zaida H et al 2014 | Bemnet A et al 2012 | Roba K et al 2018 | Masresha T et al 2019 | Adamu B et al 2015 | EPHI 2016 |
| **Selection** | 1. **Representativeness** |  |  |  |  |  |  |  |  |  |  |  |  |  |
|  | Truly representative* | * |  | * | * | * |  |  | * |  |  | * |  | * |
|  | Somewhat representative* |  |  |  |  |  |  |  |  |  |  |  | * |  |
|  | Selected group |  |  |  |  |  |  |  |  |  |  |  |  |  |
|  | No description |  |  |  |  |  |  |  |  |  |  |  |  |  |
|  | 1. **Sample size** |  |  |  |  |  |  |  |  |  |  |  |  |  |
|  | Justified and satisfactory* | * |  | * | * |  |  | * | * |  |  | * |  |  |
|  | Not justified |  |  |  |  |  |  |  |  |  |  |  |  |  |
|  | No information |  |  |  |  |  |  |  |  |  |  |  |  |  |
|  | 1. **Non-respondents** |  |  |  |  |  |  |  |  |  |  |  |  |  |
|  | Proportion* | * | * | * | * | * | * | * |  | * | * | * | * | * |
|  | Unsatisfactory |  |  |  |  |  |  |  |  |  |  |  |  |  |
|  | No information |  |  |  |  |  |  |  |  |  |  |  |  |  |
|  | 1. **Ascertainment of the exposure** |  |  |  |  |  |  |  |  |  |  |  |  |  |
|  | Registered/recorded only* |  |  |  |  |  |  |  |  |  |  |  |  |  |
|  | Personal recall and records* | * | * | * | * | * | * | * | * | * | * |  | * | * |
|  | Personal record only |  |  |  |  |  |  |  |  |  |  |  |  |  |
| **Comparability** | The study controls for the most important** | ** | ** | ** | ** | ** | ** | ** | ** | ** | ** | ** | ** | ** |
|  | No control of factors or no information |  |  |  |  |  |  |  |  |  |  |  |  |  |
| **Outcome** | 1. **Assessment of outcome** |  |  |  |  |  |  |  |  |  |  |  |  |  |
|  | Independent blind assessment** |  |  |  |  |  |  |  |  |  |  |  |  |  |
|  | Record linkage** | ** | ** |  |  | ** | ** | ** | ** | ** | ** | ** | ** | ** |
|  | non-standard or non-validated laboratory methods |  |  |  |  |  |  |  |  |  |  |  |  |  |
|  | No description |  |  |  |  |  |  |  |  |  |  |  |  |  |
|  | 1. **Statistical test** |  |  |  |  |  |  |  |  |  |  |  |  |  |
|  | Statistical test used to analyse the data clearly described, appropriate and measures of association presented including confidence intervals and probability level (p value)* | * | * | * | * | * | * | * | * | * | * | * | * | * |
|  | Statistical test not appropriate, not described or incomplete. |  |  |  |  |  |  |  |  |  |  |  |  |  |
| Total score |  | 9 | 7 | 7 | 7 | 8 | 7 | 8 | 8 | 7 | 7 | 8 | 8 | 8 |

This scale has been modified from the Newcastle-Ottawa Quality Assessment Scale for cohort studies to perform a quality assessment of cross-sectional studies for the systematic review. The star/s represents score/mark.
