# Supplementary material for: Prevalence and associated factors of zinc deficiency among pregnant women and children in Ethiopia: a systematic review and meta-analysis
Source: BMC Public Health. 2019 Dec 11;19:1663. doi: 10.1186/s12889-019-7979-3 (PMC6907210; doi:10.1186/s12889-019-7979-3)
Supplement: Supplementary file 3 — Additional file 3. Table that shows the laboratory analysis approaches used to determine serum zinc level of the study participants of the studies included in the systematic review and meta-analysis of prevalence and associated factors of zinc deficiency among pregnant women and children in Ethiopia, 2019. [file 12889_2019_7979_MOESM3_ESM.docx]

Table that shows the laboratory analysis approaches used to determine serum zinc level of the study participants of the studies included in this meta-analysis.

| Author name | Study participants | Laboratory analysis approach |
| --- | --- | --- |
| Zaida H et al 2014 | Children | A blood sample was taken via venipuncture and zinc was determined by atomic absorption spectroscopy. Zinc cut-offs were 65 mg/dl for children<10 years, and <70 mg/dl for children≥10 years as per the recommendation of IZiNCG. |
| Bemnet A et al 2012 | Children | Concentration of zinc in serum was determined using an Inductively Coupled Plasma Mass Spectrometer. The 200μl serum sample was aliquot into the Teflon tube and covered with Teflon ball. After adding 1 ml of concentrated HNO_3_ and it was heated. The cutoff value for zinc deficiency was <75ug/dl. |
| Roba K et al 2018 | Children | The concentration of zinc in serum was determined by using Shimadzu Flame Atomic Absorption Spectroscopy. Serum sample (200μl) was added into a trace metal-free plastic test tube and diluted with 6% butanol in 1:5 ratios. Zinc concentration was measured using an air-acetylene flame at a wavelength of 213.9 nm and a slit width of 0.7 nm. A cut-off value for serum zinc was 65μg/dl as per the recommendation of IZiNCG. |
| Masresha T et al 2019 | Children | The blood was allowed to clot for 30 min and centrifuged at 3000 rotations per minute (rpm) for 10 min. An aliquot was separated based on the recommended procedures of the International Zinc Nutrition Consultative Group. Serum zinc concentration was measured using Shimadzu Flame Atomic Absorption Spectroscopy with an air-acetylene flame at a wavelength of 213.9 nm and a slit width of 0.7 nm. Serum zinc deficiency was defined as concentration < 65ug/dl. |
| Adamu B et al 2015 | Children | Serum zinc was determined by using Shimadzu Flame Atomic Absorption Spectroscopy with an air-acetylene flame at a wavelength of 213.9 nm and a slit width of 0.7 nm. About 200μl of serum sample was added into a trace metal free plastic test tube and diluted by addition of 6% butanol in 1:5 ratios. Zinc deficiency was defined as a serum zinc level of less than 65μg/dl as per the recommendation of IZiNCG. |
| EPHI 2016 | Children | Zinc concentration was done using Shimadzu Flame Atomic Absorption Spectroscopy. Samples were measured in duplicate and an internal control sample was analyzed with each batch of samples. Staff serum samples were used as a control during zinc analysis every 60 samples. Serum zinc deficiency was defined as a concentration <70μg/dl for all age groups. |
| Gebremedhin S et al 2011 | Pregnant women | The sample was allowed to clot for 20 min and consecutively centrifuged at 3000 × g for 10 min. Serum zinc concentration was determined using Flame Atomic Absorption Spectrometer. Zinc deficiency was defined as a serum zinc level of less than 56μg/dl during the first trimester or less than 50μg/dl during the second or third trimester as per the recommendation of IZiNCG. |
| Stoecke BJ et al 2009 | Pregnant women | Plasma was separated from the sample within 2 hours using methods appropriate for trace minerals and stored at −20 °C until analyzed for zinc by atomic absorption spectroscopy. Plasma Zinc< 50ug/dl was used as cutoff for zinc deficiency |
| Afework K et al 2008 | Pregnant women | The concentration of serum zinc was determined using an inductively coupled plasma mass spectrometer. The serum sample (200μl) was aliquot into the Teflon tube and covered with Teflon ball. After adding 1 ml of concentrated HNO3 and it was heated. The diluted serum solution was used for analysis of the element. Deficiency of zinc was defined at their serum levels < 75ug/dl. |
| Regassa K 2017 | Pregnant women | The analysis was made using Shimadzu Flame Atomic Absorption Spectrometers. Samples were analyzed at a wavelength of 213.9nm and a slit width of 1nm.Initially; standards of 0, 0.1, 0.2, 0.3, and 0.4 PPM were prepared by diluting 1000ppm zinc standard inappropriate volume of 6%butanol.Then the standards were used for calibration. The samples were prepared by diluting well vortexes 200μl serum samples in 2 ml of 6%butanol. Zinc deficiency was defined as a serum zinc level <56μg/dl during the first trimester or <50μg/dl during the second or third trimester as per the recommendation of IZiNCG. |
| Kumera G K et al 2015 | Pregnant women | The blood was allowed to clot for 20 minutes, centrifuged at 3000 rotations per minute for 10 minutes, and sera were separated from the cells following standard procedures. Serum zinc concentration was determined using Shimadzu Flame Atomic Absorption Spectroscopy. Zinc deficiency was defined as a serum zinc level of <56μg/dl during the first trimester or <50μg/dl during the second or third trimester as per the recommendation of IZiNCG. |
| Rosalind S et al 2008 | Pregnant women | Samples were collected venipuncture samples using rigorous collection and separation procedures. Plasma zinc was analyzed by flame atomic absorption spectrophotometry and plasma zinc <76*μg*/dl was used as cutoff zinc deficiency. |
| Mekonen A 2016 | Pregnant women | The concentration of zinc in serum was determined by using Shimadzu Flame Atomic Absorption Spectroscopy. Serum samples were added into a trace metal free plastic test tube and diluted by the addition of 6% butanol in 1:5 ratios. Zinc concentration was measured using an air-acetylene flame at a wavelength of 213.9 nm and a slit width of 0.7 nm. Serum zinc level of < 56μg/dl during the 1^st^ trimesters or <50μg/dl during the 2^nd^ or 3^rd^ trimesters were used as per the recommendation of IZiNCG. |

*IZiNCG=International Zinc Nutrition Consultative Group*
