# Supplementary material for: Prevalence and associated factors of zinc deficiency among pregnant women and children in Ethiopia: a systematic review and meta-analysis
Source: BMC Public Health. 2019 Dec 11;19:1663. doi: 10.1186/s12889-019-7979-3 (PMC6907210; doi:10.1186/s12889-019-7979-3)
Supplement: Supplementary file 4 — Additional file 4. Forest plot for coffee intake and zinc deficiency among pregnant women in Ethiopia, 2019. [file 12889_2019_7979_MOESM4_ESM.docx]

**Study name**

**Statistics for each study**

**Odds ratio and 95% CI**

**Weight (Random)**

**Odds**

**Lower**

**Upper**

**Zinc**

**No zinc**

**Relative**

**Ratio**

**Limit**

**Limit**

**Z-Value**

**P-value**

**Deficiency**

**Deficiency**

**Weight**

Gebremedhin S et.al

1.385

1.027

1.866

2.137

0.033

188 / 329

182 / 371

53.95

Regassa K.

2.330

1.540

3.524

4.006

0.000

105 / 159

111 / 244

46.05

1.760

1.058

2.925

2.179

0.029

**0.01**

**0.1**

**1**

**10**

**100**

Forest plot for coffee intake and zinc deficiency among pregnant women in Ethiopia

Meta-analysis
