# Supplementary material for: Prevalence and associated factors of zinc deficiency among pregnant women and children in Ethiopia: a systematic review and meta-analysis
Source: BMC Public Health. 2019 Dec 11;19:1663. doi: 10.1186/s12889-019-7979-3 (PMC6907210; doi:10.1186/s12889-019-7979-3)
Supplement: Supplementary file 5 — Additional file 5. Forest plot for low intake of animal source foods and zinc deficiency among pregnant women in Ethiopia, 2019. [file 12889_2019_7979_MOESM5_ESM.docx]

**Study name**

**Statistics for each study**

**Odds**

**Lower**

**Upper**

**Relative**

**Ratio**

**Limit**

**Limit**

**Z-Value**

**P-Value**

**Weight**

Regassa K.

1.544

1.020

2.336

2.056

0.040

25.96

Mekonen A

2.824

1.659

4.807

3.826

0.000

21.10

Gebremedhin S et al

3.117

2.181

4.454

6.241

0.000

28.57

Kumera G et al

3.227

2.056

5.064

5.095

0.000

24.37

2.565

1.800

3.656

5.214

0.000

**0.01**

**0.1**

**1**

**10**

**100**

Forest plot for low intake of animal source foods and zinc deficiency among pregnant women in Ethiopia

Meta-analysis
