# Supplementary material for: Prevalence and associated factors of zinc deficiency among pregnant women and children in Ethiopia: a systematic review and meta-analysis
Source: BMC Public Health. 2019 Dec 11;19:1663. doi: 10.1186/s12889-019-7979-3 (PMC6907210; doi:10.1186/s12889-019-7979-3)
Supplement: Supplementary file 6 — Additional file 6. Forest plot for inadequate diet diversity and zinc deficiency among pregnant women in Ethiopia, 2019. [file 12889_2019_7979_MOESM6_ESM.docx]

**Study name**

**Statistics for each study**

**Odds**

**Lower**

**Upper**

**Relative**

**Ratio**

**Limit**

**Limit**

**Z-Value**

**P-Value**

**Weight**

Mekonen A

1.194

0.721

1.975

0.689

0.491

30.42

Gebremedhin S et al

2.373

1.716

3.281

5.227

0.000

36.95

Kumera G et al

3.222

2.068

5.019

5.174

0.000

32.63

2.128

1.283

3.527

2.927

0.003

**0.01**

**0.1**

**1**

**10**

**100**

**No zinc deficiency**

**Zinc deficiency**

Forest plot for inadequate diet diversity and zinc deficiency among pregnant women in Ethiopia

**Meta-analysis**
